# Cyanobacteria and Algal-Based Biological Life Support System (BLSS) and Planetary Surface Atmospheric Revitalizing Bioreactor Brief Concept Review

**DOI:** 10.3390/life13030816

**Published:** 2023-03-17

**Authors:** Ryan Keller, Karthik Goli, William Porter, Aly Alrabaa, Jeffrey A. Jones

**Affiliations:** 1 Center for Space Medicine, Baylor College of Medicine, Houston, TX 77030, USA; 2 College of Natural Sciences and Mathematics, University of Houston, Houston, TX 77204, USA

**Keywords:** life support, atmospheric revitalization, in situ resource utilization, space exploration, microalgae, spirulina, BLSS, ISRU, cyanobacteria, methane

## Abstract

Exploring austere environments required a reimagining of resource acquisition and utilization. Cyanobacterial in situ resources utilization (ISRU) and biological life support system (BLSS) bioreactors have been proposed to allow crewed space missions to extend beyond the temporal boundaries that current vehicle mass capacities allow. Many cyanobacteria and other microscopic organisms evolved during a period of Earth’s history that was marked by very harsh conditions, requiring robust biochemical systems to ensure survival. Some species work wonderfully in a bioweathering capacity (siderophilic), and others are widely used for their nutritional power (non-siderophilic). Playing to each of their strengths and having them grow and feed off of each other is the basis for the proposed idea for a series of three bioreactors, starting from regolith processing and proceeding to nutritional products, gaseous liberation, and biofuel production. In this paper, we discuss what that three reactor system will look like, with the main emphasis on the nutritional stage.

## 1. Introduction

Human expeditions to other celestial bodies have been achieved with the Apollo program, but current goals are to travel farther and for longer. With missions no more than 2 weeks in duration, all food, fuel, and other supplies were packed in from launch to landing with no need for resupply. The International Space Station (ISS) has enabled continuous human presence in space for over 2 decades, proving our capacity to live off-planet for an extended time frame, however, in low Earth orbit (LEO), a resupply vehicle is only a few hours away.

Some current goals of the newer solar system exploration reference missions are to travel farther away from Earth, with longer duration, and greater need for autonomous crew sustenance. Several lunar design reference missions call for a continuous presence on the moon to learn how to reliably live off the native resources [1]. Mars surface reference missions have both short and long surface stay options, but with 2–3 years total mission duration. Both would require solutions to three of the main limiting factors, food, air, and fuel. One of the critical factors in planning a mission to Mars is timing the launch within the launch window. Based on the Hohmann transfer orbit, these limited windows of opportunity typically occur once every 26 months when Earth and Mars are in suitable positions to minimize the fuel and time expended [2].

Since 1976 with the Viking program, we have been able to send unmanned spacecraft to the surface of Mars. Clearly, the current knowledge of orbital trajectories and mechanics has allowed us to reliably reach the planet with conventional rocketry, but the challenge for human missions remains the need for life-sustaining resources, and a return trip home. The question NASA and other agencies are trying to answer is how to reliably sustain human life in such austere environments, beyond immediate aid. A one-way trip to Mars involves between a 7–12 month exposure to microgravity, a lack of gaseous resources, cosmic radiation exposure, and a lack of available nutrients to harvest.

Two of the biggest limiting factors of what we can launch from Earth are mass and volume. Providing all the food, air, and fuel for crewmembers from launch will incur a heavy tax on the capacity of our craft to carry other necessary supplies. Further, any unplanned burns or the life support system failure of one of the oxygen tanks could be irreversibly terminal. This is why we must look for more sustainable options for food, oxygen, fuel, or a combination, something generated during the mission that would reduce the percentage of static material occupying volume on the spacecraft.

**Figure 1:**
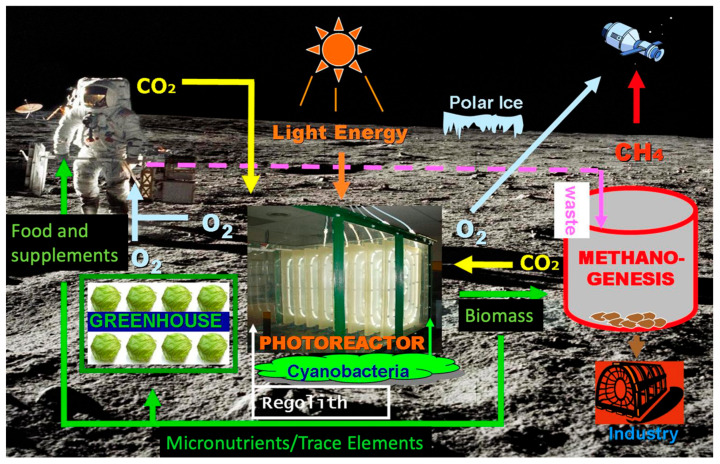
Multi-stage planetary bioreactor concept [6].

A possibility for this is a three reactor system (Figure 1). Stage 1 involves using siderophilic cyanobacteria and their organic bioacids to liberate minerals and gasses from the lunar and Martian regolith. Martian regolith is full of essential minerals, metals, and more needed for life to flourish, including iron. However, it is not available to a lot of organisms in its current state and environment. Stage 2 will involve a photosynthetic reactor using a different species of microorganism that has been studied and cultivated for human consumption here on Earth. This reactor will use the compounds and minerals liberated from the regolith in Stage 1, along with solar radiation and the carbon dioxide from the crew, with possible supplementation by the Martian atmosphere, to produce an edible biomass to supplement the food stores for the mission. Stage 3 will involve a third bioreactor with a different microorganism to form biofuels. Excess biomass from Stage 2 along with some nutrients from Stage 1, if necessary, could be used as nutrition for this reactor as it creates the desired fuel. The current optimal candidate for fuel production is methane, due to its reasonably high Isp, its incorporation into several design reference missions, and known bacterial digestive effluent, e.g., human colonic flatus.

Many teams, agencies and projects have aimed to solve this life support puzzle. Namely, the Lunar-Mars Life Support Test (LMLSTP), BioHAB, and the Micro-Ecological Life Support System Alternative (MELiSSA), all provided advances in understanding of the intricacies of developing self-sustainable life support systems. The LMLSTP aimed to incorporate higher order plants in an isolated ecological circle. The BioHAB project aimed to create a biological habitat that would provide support functions itself through a mycelium-based wall with other microorganisms included. MELiSSA is an ongoing initiative to create autonomous systems for exploration missions capable of regenerating food, oxygen, and water. The loop concept for this project consisted of four compartments (liquefying, photoheterotrophic, nitrifying, and photoautotrophic) working together to create biologically relevant compounds, as well as recycle waste. Many of the teams involved in the MELiSSA project have made large strides in bioreactor technology involving many of the principles and species mentioned in this review. A brief summary of these projects can be found in a similar review by these authors [3]. Not mentioned in that review is the Biosphere 2 program. Biosphere 2 was a project undertaken in the 1990s with the goal to create “the first indefinitely operating bioregenerative system capable of full human life support” [4]. The structure itself, which is now run by the University of Arizona, is a multi-ecosystem facility designed to be completely isolated from the outside world except for the sunlight and energy used for atmospheric temperature control. Ecosystems included were tropical rain forest, mangrove wetlands, a fog desert, savannah grassland, and an ocean with a coral reef. Two manned missions were created. The first one began in 1991 and lasted for 2 years, though many problems occurred, including medical emergencies with brief evacuations and atmospheric calculation failures resulting in very high carbon dioxide levels attributed to the high rates of respiration by soil bacteria. The second mission was cut short largely due to budgetary problems. Today, the artificial ecosystem facility serves as a model for research into other relevant topics like the understanding and mitigation of the effects of climate change [5].

In this paper, we will briefly review data surrounding Stages 1 and 3 while focusing on Stage 2, the nutrition and biogas generation component of the system. We discuss a few species of microorganisms that have been identified as candidates for this system, going into their nutrition benefits and any drawbacks. Further, we will discuss the state of the research into oxygen liberation and carbon fixation in these systems and what that may look like at scale.

## 2. Stage 1: Resource Acquisition from Regolith

Stage 1 of this bioreactor system will be an ISRU reactor to facilitate the conversion of the native regolith to available substrates for the Stage 2 reactor. As will be discussed later, our photosynthetic reactor needs substrates in a medium to grow, and targeting specific components and concentrations within that medium optimizes production. When deploying these reactors to the moon, Mars, and beyond, it becomes necessary to harvest these substrates and minerals from the destination environment. Verseux et al. conducted a review of the experiments determining the composition of the Martian regolith and found that all of the elements needed for cyanobacterial and microbial growth (C, H, I, N, P, S, K, Mg, Na, Ca, Fe, Mn, Cr, Ni, Mo, Cu, Zn, etc.) are present, the remaining task is making them available for use [7]. Siderophilic (iron-loving) cyanobacteria (CB) are ancient organisms that have the ability to process regolith using a combination of many organic acids. These acids have the ability to dissolve rock, including the basalt rocks that are prevalent on Earth, the moon, and Mars [6,7]. Organic acids work as chelators that transition the elements in the regolith to the liquid phase for availability [7]. For example, iron can be chelated out into a dissolved form and be recovered by electrolysis. Water that is formed can be split into oxygen and hydrogen gasses. The resources present but molecularly out of reach are suddenly available to us.

**Figure 2:**
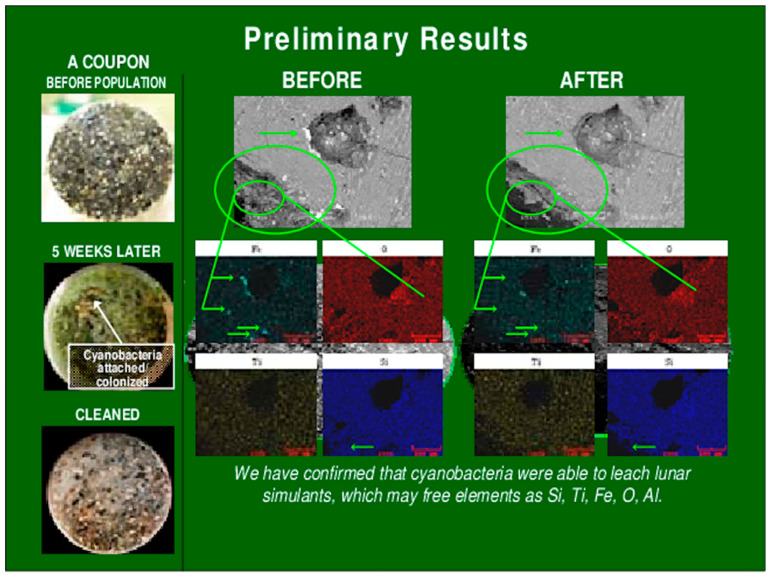
Stage 1 concept: Bioweathering of Minnesota basalt grains (lunar soil simulant) by siderophilic CB JSC-12 [6].

It was shown that when producing desirable compounds, CB are 24 times more efficient than traditional agriculture and that purifying these acids is conducted using a phase separation technique [8]. This concept was carried out in a laboratory setting and is in line to be developed into a modular factory [8]. Brown et al. demonstrated the bioweathering characteristics of different species of siderophilic cyanobacteria. They showed that CB growth was stimulated when presented to their lunar and Martian regolith analogs and that it stimulated the production of 2-ketoglutaric acid [6]. Through trials, that team continued on to reveal a strain, named JSC-12, that demonstrated higher intrinsic bioweathering capability than the other tested organisms. Their visual results showing the bioweathering activity of JSC-12 are provided below (Figure 2). This strain is promising as a candidate to accompany missions to harvest regolith, especially after the demonstration on analogs.

Siderophilic CB, as opposed to non-siderophilic CB, are substantially more effective at growing in iron replete environments like Mars. In fact, when compared to *Synechococcus* sp. *PCC 7002*, *Leptolyngbya JSC-1* cells had over double the PSI:PSII ratio (1.8 to 4:1, respectively) [9]. Seeing this distinction between the growth rates of siderophilic and non-siderophilic CB (of which our nutritive species belong), along with the fact that non-siderophilic CB do not produce the same degree of organic acids, demonstrates the need to have another species function in breaking down the regolith. Zarrouk’s medium, which is an invented medium designed for optimal CB growth, is made up of elements that can all be found in the regolith and can be released by the siderophilic CB. Starting with an ISRU bioreactor for Stage 1 will introduce the continuous supply of the elements needed to keep our Stage 2 reactor growing at an acceptable rate. More on the bioprocessing of regolith and the release of important elements can be found in a parallel paper by Dr. Igor Brown titled, “*Siderophilic Cyanobacteria to Link the Biological In-situ Resources Utilization (Bio-ISRU) and Closed Biological Life Support Systems (CBLSS)*”.

A challenge of using native regolith is the presence and abundance of perchlorate salts ubiquitously spread throughout Mars. In excess, perchlorates are known to harm the metabolic pathways of terrestrial organisms, including various microorganisms, and can amplify the deleterious effects of UV radiation. According to data gathered from various locations on Mars, perchlorate levels are 0.4–0.6 wt%, with some values as high as 2 wt%, which is three to four magnitudes greater than the conditions on Earth. A recent study, which tested the effects of exposing 0.25 to 1% magnesium perchlorate to 17 various strains of cyanobacteria over 14 days, resulted in lipid peroxidation (an indicator for oxidative stress) and at least partial growth inhibition [10]. Although growth was significantly inhibited compared to their controls, *Chroococcidiopsis thermalis*, *Arthronema africanum*, and *Leptolyngbya foveolarum* continued to display growth at the highest magnesium perchlorate concentrations [10]. When under perchlorate exposure, *C. thermalis* and *L. foveolarum* also exhibited a respective 40–60% increase and a 25% increase in carotenoid contact, demonstrating a form of protective response. Carotenoids are known to mitigate oxidative stress by inhibiting free radical reactions [10]. Another similar study exposed strains of *Chroococcidiopsis* sp. to various levels of Mg-, Na-, and Ca- perchlorates over a 55-day period. The study showed a progressive decrease in the growth as the perchlorate concentration was increased and at 300 mM of potassium perchlorate no growth was exhibited [11]. Both studies cited the tolerance to perchlorates as at least partially related to the cyanobacteria’s antioxidant capacities. A solution opposite to the building up of defenses is the removal of the offender. Some proposed ideas on mediating the toxicity of perchlorates include the introduction of organic materials, perchlorate volatilization, phytoremediation, ferric chloride, or hydrochloric acid to breakdown perchlorates as needed [10,12]. Another is to use the existing biochemical pathways of perchlorate reducing bacteria to decompose perchlorate into Cl- and O_2_ [13]. Further studies should investigate the innate defense mechanism of various species and the application of chemical and biological neutralization to open up more bioengineering possibilities.

## 3. Stage 2: Biomass Production (Nutrition)

Macronutrient proportions are near the top of the list of useful properties of a microorganism for consumption. The goal is to fulfill many of the macro- and micronutrient needs of humans as a supplement to the normal food supply. Many analyses of nutrient concentrations of different species have yielded an understanding that the vast majority of their dry weight will be protein with carbohydrates and fats lagging behind, not including their ability to provide a wide array of minerals, vitamins, amino acids, and fats to their consumers.

**Figure 3:**
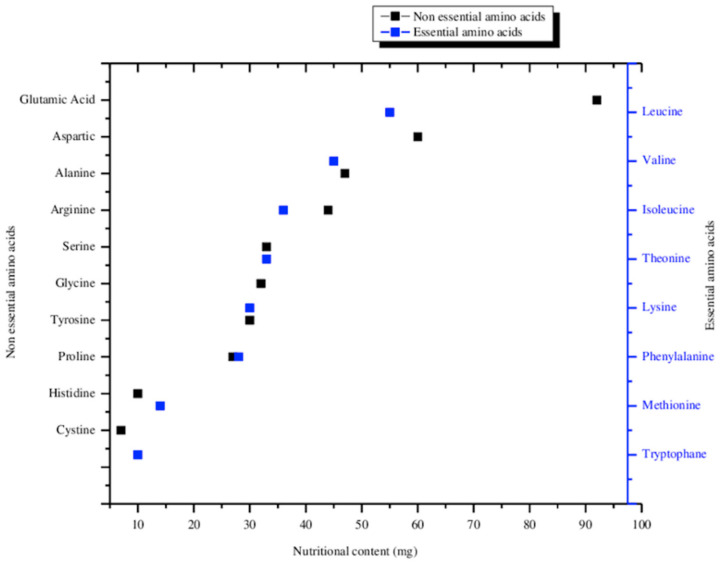
Essential amino acid content for Arthrospira [14].

For reference, the essential amino acid requirements for adults can be found in Table 1. Given that *Arthrospira*, for example, provides 10 mg/g of histidine, crewmembers must consume at least a 1:1 ratio of grams of *Arthrospira* to their body mass in kg (Figure 3) [14]. Every other amino acid produced by *Arthrospira* is present at relative concentrations higher than histidine, making it the limiting factor. Since astronauts typically range in body mass from 50–95 kg, this is not a huge burden on their diet, especially if algal biomass is made a major contributor. *Chlorella vulgaris* contains an overall lower concentration of amino acids than *Arthrospira* [15]. Specifically, histidine concentrations are 50% of those in *Arthrospira*, providing a challenge for matching the efficient incorporation into diets. However, doubling the daily consumption of CB to offset this should not be a major burden either, considering 100 g of food is comparable to the size of two eggs. Studies of other species have been carried out but are minimal. Caution should be exercised when burdening crewmember diets with too much algal biomass however, as there are possible psychological implications when pulling people too far away from enjoyable foods.

Essential fatty acids can also be provided by a diet of microorganisms. Omega-3s (alpha-linolenic acid (ALA), eicosapentaenoic acid (EPA), docosahexaenoic acid (DHA)) and omega-6s (gamma-linolenic acid (GLA), arachidonic acid (ARA)) are present to some degree in many microalgal species, just in different proportions. Two of the species that we will be discussing further, *Arthrospira* and *Chlorella*, were analyzed for specific lipid content. *Arthrospira* showed around a 20% lipid content of GLA and 17% from linoleic acid, but mostly undetectable levels of ALA, EPA, ARA, DHA, and others [17]. *Chlorella* showed about 15% lipid content from ALA and 12% from linoleic acid, but also near undetected for GLA, ARA, EPA, DHA, and others [17]. There are mixed results for these fatty acid levels as some studies have been able to detect DHA only in some strains of *Arthrospira and Chlorella*. *Lang* et al. ran an analysis of the fatty acid profiles of 2076 microalgal strains in the collection at Göttingen University. They found that different species specialized in creating different essential fatty acids. DHA is prevalent in Dinophyta, Haptophyta, and Euglenoids, with the highest content in *Ceratium horridum* at 29.3% of lipids [18]. EPA was found to some degree across all the groups studied with frequency highest in *Eustigmatophyceae*, *Glaucophyta*, *Xanthophyceae*, and *Rhodophyta* (highest at 52.4%) [18]. In their study, one *Chlorella* strain showed 24.2% EPA content, conflicting with the earlier study noted. Arachidonic acid is prevalent in Phaeophyceae, but Euglenoid strains have shown relatively higher levels at above 5% [18]. There is not one single perfect organism to supply all the essential fatty acids. Therefore, other supplementation or genetic alteration might have to be explored.

To stress the importance of these FAs, DHA has been found to play an important role in maintaining brain health in adults beyond its developmental role in children. Among the benefits, DHA reduces the development of unipolar depression, multiple sclerosis, and partially cognitive decline [19]. The mechanism is suspected to be the inhibition of apoptosis by sphingosine through the inhibition of types of phospholipase A2 [19]. Coronary artery disease is also prevented through a decrease in the total cholesterol to HDL ratio [19]. Asthma exacerbation, cancer cell proliferation, inflammation, and platelet aggregation were all decreased because of supplementing diets with DHA and EPA [19]. Fish oil supplements contain both DHA and EPA, with EPA generally occupying a higher content. Our bodies can make EPA and DHA from ALA. Specifically, 8–20% of ALA is converted to EPA, while 0.5–9.0% is converted to DHA at any given moment [20]. Given that fish oil has an EPA to DHA ratio of at least 4:1, the human conversion mechanism can give us a higher relative DHA level but may not be as efficient at ingesting a set amount of preformed EPA and DHA.

While most of the information surrounding microorganisms for nutrition is positive, there are a few documented drawbacks. Consuming *Cellulomonas*, with a protein content of 80%, could lead to high uric acid levels and predispose people to conditions like gout or uric acid stones [21]. Another reason some microorganisms are not used for widespread consumption is the potential for creating toxins, including microcystins, nodularins, saxitoxins, and anatoxins [22]. The goal should be to find species that do not produce these toxins so as to not put the crew at risk of dangerous reactions. Such microorganisms include some species of the genera *Arthrospira*, *Chlorella*, *Euglena*, and *Dunaliella* [23].

### 3.1. Arthrospira 

*Arthrospira* is a genus of algae that has been well studied for its nutritional content for years. It is believed to have been consumed in Africa and by the Aztecs in Mexico, while today it has been studied as a solution for malnutrition across the globe and its extensive enzymatic benefits beyond simple nutrition [21,24,25,26]. In simple numbers, for every 100 g after ideal growing conditions, *Arthrospira* contains over 350 calories, over 4 g of fats, over 17 g of carbohydrates, and over 60 g of proteins [22,23,25,26]. Due to the relatively low fat content, it is recommended that a diet of *Arthrospira* be supplemented with seeds, nuts, and fish for another source of omega-3 fatty acids [22]. However, the fats that *Arthrospira* does contain are essential ones, including linoleic and linolenic acid [21]. Further, because the WHO recommends at least 55% of the average person’s daily energy come from carbohydrates, the carbohydrate content in analyzed *Arthrospira* cultures will not be enough to fulfill a proper diet [21]. Vitamins are abundant in *Arthrospira*, containing A, B1, B2, B3, B6, E, H, folacin, pantothenic acids, inositol, and enough B12 in 1 g of dry weight to fulfill your daily requirement [21]. It grows at higher alkalinities (ideal pH 10.5) making it less susceptible to pathogens than other species [23,27]. 

*Arthrospira* is usually grown in solutions containing KNO_3_ or NaNO_3_, but was also shown to use urea as a source of nitrogen [23,27]. Since humans excrete mostly urea compared to other nitrogen sources, this could prove a key nitrogen recycling point between crewmembers and bioreactors. However, urea does damage photosystem II in high concentrations and is also known to off-gas as ammonia in alkaline environments like the one *Arthrospira* prefers [23]. Iron is an important element in *Arthrospira* as a limiting factor for pigment formation [28] and in humans for hemoglobin formation. Even in low iron conditions in the environment, *Arthrospira* still concentrated large amounts of the metal [27], making it a valuable asset for iron delivery to crewmembers. Considering that the Martian regolith is composed of a large amount of iron, there is plenty of material to harvest and concentrate for consumption. 

To touch on other benefits of *Arthrospira*, Torres-Duran et al. explained that the organism has some hepatoprotective properties. When introduced into a situation where hepatocyte injury was elicited from CCl4 administration, *Arthrospira maxima* lowered the aspartate aminotransferase (AST) levels and altered the quality of triacylglycerols (TAGs) [29]. The mechanism is believed to be related to the bacteria’s ability to synthesize and release nitric oxide (NO) and antioxidants, including carotenoids and phycocyanins [29]. Cho et al. gathered that many phenolic compounds are present in *Arthrospira*, including chlorogenic acid, synaptic acid, salicylic acid, trans-cinnamic acid, and caffeic acid [30]. Those combined with the presence of phycobilins and phycocyanins give anti-inflammatory, antiviral, antioxidant, antithrombotic, vasodilatory, antidiabetic, neuroprotective, hepatoprotective, and anticarcinogenic properties to *Arthrospira* [23,30]. Concentrations of these compounds are directly related to the concentration of nitrogen in the growth media [31].

With regards to toxin production, long-term studies were conducted on rodents to see if there were any negative effects of diets with *Arthrospira* as a major contributor. Diets composed of 30% *Arthrospira* showed no evidence of acute, subacute, chronic, reproductive, or genotoxic effects [25]. The only adverse effect seen was in one trial where a 60% *Arthrospira* diet elicited an increase in kidney, heart, and lung weights and a nephrocalcinosis syndrome in mice [25]. While species of *Arthrospira* do not produce toxins themselves, there have been records of some cultures that had contamination with anatoxin A and microcystin [22]. This is thought to be due to unmonitored cultivation but does point to the importance of isolating pure cultures of the desired species.

### 3.2. Euglena

*Euglena gracilis* is another microorganism with particular interest as a diet replacement and supplement. Its macronutrient content ranges from 39–61% of protein, 14–18% of carbohydrates, and 14–20% of fats [32]. Like *Arthrospira*, it is a rich source of proteins, is high in methionine, contains all the essential vitamins, and many important fats [33]. Aemiro et al. studied the effects of replacing a soybean diet with *Euglena* in sheep, finding reduced energy loss through methane release and a reduction in total crude protein loss (increased crude protein efficiency), while exhibiting no change in the average daily body weight gain [34]. One problem with cultivating *E. gracilis* is the noted higher tendency than other species to be preyed upon by other organisms through contamination [33]. This can be fixed by its ability to grow in acidic environments (pH 3.5) [33]. This species is not as studied as others but has a similar nutrition profile. Due to other species, namely *Arthrospira*, having been more heavily described and used commercially, unless a substantial benefit distinguishes this organism from others, solutions should be directed elsewhere. 

### 3.3. Chlorella

*Chlorella* was first mass produced by the Taiwanese in the 1960s and adoption ramped up from there [24]. However, production of this organism has not taken as big of a share in the market due to the adoption of *Arthrospira* in more circles for its alkaline growing environment [24]. Compared to *Arthrospira*, *Chlorella* on average contains less protein, more lipids, and fewer carbohydrates [35]. Strains of this organism were shown to contain 57% protein, 6% carbohydrates, and 17% lipids [36]. Even though the total fat percentages are higher for this organism, it is important to see that the levels of important fats, including stearic acid, linolenic acid, and arachidonic acid, were far lower than *Arthrospira*, while the levels of docosahexaenoic acid were far higher [35]. *Chlorella* cultures also contain the same minerals as *Arthrospira*, with the exception of Cd, but at different concentrations (Fe levels over twice the levels in *Arthrospira*) [35]. In an experiment where a strain of *Chlorella* was tested in a nitrogen limited environment, the organism changed its composition, increasing the carbohydrate proportion to 65% (mainly starches) and the protein concentration to 20% [37]. However, another review showed that in a nutrient deficient medium, the organism only exhibited an increase to 10% carbohydrate content [29]. *Chlorella* cannot be harvested by regular filtration and must be harvested by micro- or ultrafiltration, a process much more demanding and expensive [38]. Alternatively, centrifugation was mentioned as a possible alternative for harvesting, as it yields faster results than traditional heat drying of the biomass [38].

### 3.4. Dunaliella

*Dunaliella* is another microorganism that has potential nutritive power. It grows in extremely salty environments (6–12%) and can be grown in large cultures [24]. It is easily digested by humans due to its lack of cellulosic cell walls [39]. Standing out from the other species, it contains a very high carotenoid content, even boasting the highest natural carotenoid concentration of all plants and algae [40]. Like the other species of interest mentioned above, *Dunaliella* has a high protein content with a low carbohydrate component. However, there has not been many analyses into the exact composition to yield any reliable numbers. Rather than being used as a nutritional supplement, *Dunaliella* is used pharmacologically, being used for hypertension, delaying atherosclerosis, Crohn’s disease, photo damage, and bronchodilation, among others [40]. Like other CB, under nitrogen deficient environmental strain, it will shift towards increased carbohydrate storage [38]. Unlike other species, the ideal pH environment for the *Dunaliella* species ranges from 7.5–8.5 [39]. This could increase contamination in any culture. Overall, *Dunaliella* should be studied more before being offered as a total solution to picking a species for bioreactors, however, the high carotenoid content with its pharmacologic and anti-oxidative properties do suggest a benefit to incorporating it into the diet of crewmembers, even if it is just supplementing a small portion of their diet.

## 4. Optimizing Biomass

Optimizing biomass production is necessary to have a real impact on mass and volume restrictions. There have been many experiments searching for solutions. In *Synechocystis* organisms, it was shown that overexpressing genes encoding for NAD(P)H:oxygen oxidoreductase (Flv3 gene) led to a 30% relative improvement in biomass production, while overexpression of carbon transporters, BicA and SbtA, in CCM increased the yield by 50–100% [26]. Exposure to gamma radiation exhibited a beneficial effect on biomass production as well. Exposure of *Arthrospira* with up to 2.0 kGy showed increases in growth and cellular components, while a reduction of the same was exhibited with exposure greater than 2.0 kGy [28]. 

Delrue et al. further examined different mediums for ideal biomass production of *Arthrospira* and found that Zarrouk’s medium (ZM) is the ideal base and that increasing iron concentrations to more than 2 mgFE/g improves nutritional quality [41]. The FloraNova medium was also found to be a suitable medium that can be dilated without significant loss in production [41]. Of note, it was also determined that ZM could be diluted up to five times before there was any impact on production up to 21 days after inoculation. Even then, diluting it to 50% showed almost no change in production from the pure ZM [27]. This is good news for production in flight and on the ground as there would not be a need to have as large a reserve of the medium or produce it at as large of a scale. The information about the nutritional improvement with increasing iron concentrations is beneficial, as well due to the high concentration of iron in Martian regolith.

Photosynthesis naturally needs light as its energy source. Therefore, ideal light levels must be determined for optimal biomass production. On traverses to the moon or Mars, solar energy is in abundance assuming window access to solar exposure for the bioreactor. The lunar surface will have its own light cycles aside from the permanently shaded regions of many craters around the poles. The solar radiance values on the Mars surface varies based on atmospheric dust concentration. Like plants, CBs and some other microorganisms have cycles where they need a break from the light in order to optimize growth. *Arthrospira* displays a level of photoinhibition, caused by photon flux disrupting photosynthesis, resulting in a reduced rate of biomass production [23]. Large cultures in bodies of water on Earth have evolved a self-shading ability as a countermeasure, but this ability may not translate to microgravity [23]. Solar radiation is not the only possible source of light for these colonies. Light emitting diodes of different wavelengths were tested to optimize biomass production in *Arthrospira*. The benefits of various wavelengths have been documented, but there remains a lack of consensus in the literature [23]. Some results indicate that red and pink light lead to the greatest increase in biomass production, blue light leads to the highest concentration of chlorophyll and phycocyanin, and green and white light lead to the highest concentration of protein [23].

With optimization of production comes the need for proper storage of excess biomass. *Papalia* et al. explored the effects of different storage techniques on concentrations of important molecules in *Arthrospira platensis*. Frozen, freeze dried, and oven dried storage were tested against fresh biomass. The results displayed drastic drop-offs in different pigments, proteins, and antioxidants for each method (Table 2) [31]. The increases in some available biomolecule levels are thought to be due to membrane degradation [31]. To note another factor in the drying process, it was shown that the more surface area that was exposed to oxygen, the less dehydroascorbic acid is left functioning in the harvest [31]. While frozen *A. platensis* seems to have prevented the most loss of the bioavailability of these molecules, the drawbacks are the cost and large power requirement [31]. For planetary-based reactor systems, the vast temperature swings, and cooler shaded areas, could naturally complement cold storage technologies. Otherwise, with the limited power generating capabilities and the mass restrictions per flight, it might be necessary to settle for a different method.

## 5. Radiation Protection

Radiation exposure is a known risk factor for spaceflight outside of Earth’s geomagnetosphere, both from galactic cosmic radiation and solar particle events. Bringing a bioreactor on-board forces the consideration of the protection from the inevitable exposure for this other organism. Having evolved before Earth’s modern atmosphere, and in polar regions with diminished magnetic field protection, many microorganisms have developed mechanisms to combat damage caused by harmful radiation. Phenolic compounds are present in many CB and algae and are important in the protection against free radical and radiation damage. Radiation protection should not only be considered for an active organism, but also for its continued protection in storage. In the same study referenced previously for comparing the types of storage, they also looked at the presence of phenolic compounds in *A. platensis* stored through different means. The table below from their paper shows that each storage method retains most of the phenolic compounds, but differ on the ones that they are missing with the highest free radical scavenging activity present in the fresh and freeze-dried biomass (Table 3) [31]. 

Complementing innate radiation protection with the available resources at intended destinations is a possibility and many teams have proposed using soil from other planetary bodies to insulate habitats and equipment. Historically, it has been understood that the greatest reduction in radiation dose occurs through the first 15–20 cm of Martian regolith shielding [42]. Llamas et al. calculated that the Martian regolith can reduce primary particle radiation by 41% at a depth of 1 m [43]. Building on this, a novel Martian regolith, the hydrogen-rich polymer brick shield concept has also been introduced, which would decrease the effective dose crewmembers receive on the Martian surface to only 123 mSv for a 500-day mission [44].

More immediately though, lunar shielding is also relevant. Unlike the Martian regolith, the lunar soil is naturally exposed to higher doses of radiation due to the lack of an appreciable atmosphere. One study demonstrated that the lunar regolith and simulants possess the ability to stop many protons in particle events. Specifically, at a density of 1.9 g/cm^3^, 5 cm of regolith stops 100 MeV protons, and 18 cm stops 200 MeV protons [45]. Another study looked at utilizing the lunar regolith with and without supplemental materials in the creation of a shielded habitat. Using NASA’s OLTARIS tool, the authors calculated that for a habitat to have an exposure kept under 150 mSv for a typical 180-day mission, it would need 160 g/cm^2^ of highlands regolith shielding [46]. They then discuss the required depths of regolith at varying densities complemented with different materials to achieve this, namely, 100 cm at nominal density of 1.6 g/cm^3^. With the demonstrated effectiveness of the lunar regolith for radiation protection, selected microorganisms could lack some of the protective qualities of their counterparts, while also being artificially shielded without requiring all shielding materials to tax the mass and volume during mission transit.

The presence of antioxidant properties in the microorganisms of interest would be an added layer of protection to crewmember health as well. B-carotenes, phycocyanins (water soluble blue pigments), superoxide dismutase, catalase, thiol systems, and other peroxidases are focuses for inclusion into the defenses of our bioreactors and the diets of crewmembers [47,48]. These molecules are both involved in light harvesting reactions and also possess antioxidant properties that scavenge free radicals, singlet oxygen, reduce cell DNA damage, and have a role in inhibiting lipid peroxidation [31,48,49]. Phycocyanins were found to be protective against hemolysis from peroxyl radicals acting on human erythrocytes [48]. Thaakur et al. explained that *Arthrospira* produces B-carotenes, phycocyanins (c-phycocyanin and allophycocyanin, among others), vitamins C and E, SOD, and selenium, which make it a powerful antioxidative organism in their scope for neuroprotection in ischemic reperfusion injury patients [22,30,48,49]. Even though certain species produce these antioxidant compounds, finding an optimal strain is necessary as qualities can vary. An analysis of two *A. platensis* strains, one from Algeria and the other from Chad revealed that the Algerian strain averaged 38% more B-carotene in its dry weight than the others (5.5 mg/g versus 4.0 mg/g respectively), demonstrating the importance of the difference in the source of the species tested [22].

Gamma radiation is used to clean fruits and vegetables after harvest and its effect on the different species of interest has been studied. For *Arthrospira* under gamma radiation, some vitamin production (including A, K, and some Bs) was stimulated by gamma rays up to 2.0 kGy, while others including thiamin (B1) and pyridoxine (B6) were shown to be sensitive to this radiation, losing 35% and 3% production quantities, respectively, at low dose radiation [28]. For minerals, Shabana et al. showed that the *Arthrospira* content of nitrogen and the activity of N-assimilating enzymes increased with increased gamma dosage up to 2.5 kGy and 2.0 kGy, respectively. Phosphorus, sodium, potassium, calcium, magnesium, and iron increased with radiation doses up to 2.0 kGy by 113%, 25%, 56%, 150%, 94%, and 400%, respectively [28]. Beyond these radiation limits, the photosynthesis-II (PSII) chain sustains damage through decreases in the allophycocyanin and phycocyanin levels [47]. Badri et al. explains this phenomenon and the entire radiation defense of *Arthrospira* in more detail, but the basic mechanism is that PSII biosynthesis genes are downregulated, while protease transcription is upregulated. In *Arthrospira*, high doses of radiation have caused the upregulation of the chromophore proteins involved in photosensing and phototaxis regulation, but the downregulation of the pilin genes for swimming motility and the gvp gas vacuole genes for floating motility [47]. This was probably developed to allow for the organism to move itself out of the path of damaging radiation. 

Exposure to full solar radiation (UV-A, UV-B, PAR) was tested for *Arthrospira*. Though this organism has antioxidant and anti-radiation damage properties as stated before, full UV exposure at temperatures regarded as its optimum environment, showed damage becoming visible after 8 h [23]. Granted, adaptations are made in the form of tightening helices to become more tolerant of light, but the fact remains that unless some type of UV shielding is put in place or the reactor is grown under artificial light conditions, there will be the possibility of damage to these cultures [23]. Thus, the microorganisms of interest not only appear to be able to protect themselves from elevated radiation exposure, but consuming them may elevate a crewmember’s level of protective antioxidants, free radical scavengers, and desmutagens (personal communications with Dr. Jeffrey Jones, M.D.).

## 6. Oxygen Production

Oxygen production and release by photosynthetic organisms complements CO_2_ fixation. The utility of photosynthetic oxygen production by various CB species has been speculated upon, as O_2_ is a critical resource for human respiration and fuel oxidation [38]. Analyses for Martian ascent vehicle strategies in DRA 5.0 were all created assuming an oxidizer would be created through ISRU, reducing the lander delivery mass by 60% [1]. While O_2_ production could be performed using physicochemical methods (i.e., by processing regolith, water, and/or atmospheric CO_2_), bioreactor-based oxygen production may demonstrate superior energetic benefits or could otherwise provide a safe redundancy that would complement physicochemical methods [50]. As organisms can serve multiple purposes for BLSS, resources do not necessarily need to be specifically dedicated towards O_2_ production, which can instead be a secondary benefit of other uses, such as food production [7].

Oxygen evolution rates under different sets of conditions are now being computed for various cultures. One study measured the oxygen evolution rates in *Synechococcus elongatus* cultures grown in a shaker incubator. Unlike organisms in the environment which are subject to light and dark cycles and other natural cycles, these cultures were grown under controlled conditions, including continuous illumination. The peak oxygen evolution rate observed was in the range of 0.38 to 3.5 mg/L/h, depending on the growth conditions [51]. Another study measured specific oxygen production rates in the species *M. aeruginosa* cultured under light–dark cycles on a solid agar plate and later in a liquid medium. The average oxygen production rate for this species was about 49 fmol/cell/h [52]. This contrasts with 25–400 fmol/cell/h for *C. vulgaris* and 30–120 fmol/cell/h for *Chlorella kessleri* [53,54,55]. The conditions for oxygen production in a photobioreactor remain to be optimized with respect to the illumination duration and timing, pH, incubation with shaking, nitrogen source, low pressure environment, and other factors [56].

On average, a single person will consume 1 kg of O_2_ per day for respiration, assuming 2 h a day of intensive physical exercise. If this oxygen demand is met photosynthetically, the required input would be 1.3 kg of CO_2_ a day per crewmember. About 1 kg a day of CO_2_ could be recycled from the respired CO_2_ of a single crewmember. Considering only the O_2_ requirements here for a total of four crewmembers (as CO_2_ requirements will be discussed later on), the whole crew will require on average 4 kg of O_2_ per day.

Under microgravity, the liquid–gas interface of a bioreactor will become more dependent on surface tension combined with several other outside forces. The process of separating evolved oxygen and other compounds of interest requires advanced technologies that are not dependent on gravitational forces. Fili et al. ran a trade-off analysis on liquid–gas separation techniques using parameters and data from the MELiSSA studies to determine the most promising technologies for efficient phase separation [57]. Out of nine techniques analyzed, they determined that passive–static capillary-based and acoustic separation were the top two. Capillary based technologies rely on container geometry and fluid properties to allow liquids and gasses separate. The acoustic technology involves piezoelectric transducers creating acoustic waves through a medium, causing air bubbles to travel to a specified node. The authors of that analysis also mention this concept passing a feasibility report for MELiSSA, as well as being currently investigated for separating vapor bubbles in cryogenic tanks in microgravity. These strategies still need further application and testing before any reliance can be assumed.

Liquid chemical rocket propellants, such as hydrazine (N_2_H_4_), may be employed as both a monopropellant and bipropellant when mixed with an oxidizer, such as liquid oxygen. A liquefied methane–liquefied oxygen (LOX) combination is currently selected as an effective ascent propellant based on the propulsion performance and potential for in situ production. About 30–40 T of methane–oxygen propellant is needed for a Mars ascent vehicle to lift itself into orbit [58]. Although methane was previously believed to be only produced by methanogenic archaea, such as *Methanobacterium thermoautotrophicum*, CB living in various environments have recently been shown to produce methane at substantial rates under various conditions of light–dark cycles [59]. Hence, CB may largely be sufficient to fulfil the role of producing the needed methane–oxygen propellant.

According to a 1:3 fuel to oxidizer ratio for a Mars ascent vehicle whose dry mass is 106 kg, the 955 kg of methane–oxygen propellant will have 716 kg O_2_. This quantity of O_2_ can be met with 90 L of cyanobacterial culture, according to the oxygen evolution rate discussed earlier. 

In conjunction with harvesting O_2_, an equally important consideration is the efficient storage of oxygen, able to be supplied as low-pressure gaseous oxygen for human respiration or as a fuel oxidizer. The bioreactors chamber could be directly connected to a compressor that separates oxygen from any carbon dioxide, hydrocarbons, or other gases and compresses the oxygen into high-pressure storage. Additionally, if liquid oxygen is desired, the compressed oxygen could be further channeled into a separation unit that uses either cryogenic distillation or noncryogenic selective adsorption processes to purify liquid oxygen. Both high-pressure oxygen and liquid oxygen are space conserving methods for oxygen storage. Such methods are currently being optimized for cost and power usage, which is an important consideration for a BLSS [60]. Importantly, liquid oxygen continues to be the most efficient method of oxygen storage for fuel oxidation [61,62,63].

Oxygen storage could certainly involve these mature technologies, namely oxygen concentrators and cylinders that are commonly used for medical and other purposes. However, given that microgravity and other planetary surfaces are low-resource environments where the supply of electricity for such technologies may be unreliable, it is also important to consider alternatives. For example, a FREO_2_ low-pressure oxygen storage system that was recently developed for medical oxygen delivery and tried in a low-resource setting, successfully continued to function under the circumstances of power outages and maintenance during a 3-month period [64]. A single one of these systems, for which the cost was USD 2300, stored and delivered an uninterrupted oxygen supply of 8 L per minute to four individuals during the 3-month period [65]. Other such systems that differ in size and number of components, oxygen storage pressure, and response to a lack of power are worth exploring [66]. A similar oxygen storage technology may appropriately be adapted for a BLSS.

## 7. Carbon Dioxide Scrubbing

The use of cyanobacteria and other microorganisms toward scrubbing carbon dioxide from the atmosphere is not a new concept. Many environmental researchers have looked to ancient autotrophs as a carbon fixation solution for the rise in atmospheric carbon dioxide over the past centuries. Similarly, as the human population increases and demand for nutrition rises, many have looked to use CB and algae for a solution. Many have sought to use these terrestrial solutions to solve problems of long-term space flight. Aside from the already discussed release of oxygen for inhabitants of a closed life support system, we must also consider the exhaled carbon dioxide of the inhabitants of such a system.

The atmospheric control of carbon dioxide must be tightly regulated for human space travel [67]. Bioreactors represent a possibly elegant solution to this problem as well as nutrition for crewmembers. Currently, studies have investigated the use of CB bioreactors for recapturing carbon dioxide. However, such bioreactors achieve varying levels of carbon dioxide consumption. Martins da Rosa et al. achieved a maximum carbon fixation rate of 0.105 g/L/day carbon dioxide [68]. Matsudo et al. maintained a tubular photobioreactor, and achieved between 0.86 and 1.7 g/L/day of carbon recaptured with steady state values of up to 1.7 g/L/day of CO_2_ achieved during steady state [69]. However, with the optimization of the bioreactors around the goal of carbon dioxide scrubbing from the atmosphere, Lopez et al. achieved carbon fixation of 1.45 g/L/day using 0.5 L flask reactors through variations of light conditions [70]. In most studies, CO_2_ is injected into the reactor in order to maintain the pH as the byproducts of cyanobacterial growth alter the solution pH. This would necessitate the use of existing carbon dioxide capture to scrub and concentrate the atmospheric carbon dioxide for injection.

Important takeaways from these studies are that a higher intensity in light correlates with a higher CO_2_ consumption rate [69,70]. Below a threshold of light intensity, *Arthrospira* will begin respiration and have a net production of carbon dioxide. For this reason, the light intensity must be maintained above a certain level and the density of the biomass must be kept beneath a certain optical density to ensure adequate light exposure [71]. 

Similar optimization reviews, such as that performed by Kumar et al., have shown that temperature impacts the solubility of various gasses and the ability of the cyanobacterial enzymes’ affinities for CO_2_ [72]. This has implications as engineers and scientists assess future bioreactor designs and decide between batch, semi-batch, or a continuous reactor design, as well as operation parameters. Zhang et al. found that by reducing the CO_2_ aeration rate, the carbon dioxide utilization efficiency increased [73]. 

Many have even looked toward altering the carbon uptake pathway of CB to increase the carbon capture. A common target of such genetic engineering is the Ribulose-1,5-bisphosphonate (RuBisCo) enzyme that helps to increase the CO_2_ concentration within the organism. While this is a critical enzyme, it has a very slow turnover rate and limits the carbon fixation. As such, many biologists and bioengineers have sought to use CB as a platform to modify and improve the efficiency of bacterial photosynthesis [74,75]. 

If we assume an average rate of expiration of 20 breaths per minute and a tidal volume of 500 mL, the average exhaled mass of carbon dioxide per person can be estimated to be between 0.7–1 kg. Utilizing 1 kg CO_2_/day/person allows for an engineering factor of safety especially with crew exercise imposing a larger CO_2_ load placed on the system. Thus, for a crew of four, the bioreactor would have to be capable of removing 4 kg of carbon dioxide on a daily basis. Thus, with current cyanobacterial bioreactors able to capture at best 1.7 g/L/day, the reactor would need upwards of 2000 L functioning constantly. As stated earlier, mass and volume are limiting factors for any space voyage and are especially restraining for any missions where terrestrial resupplying is impossible. There also remains a gap in the knowledge on how the effects of the environment outside of the near Earth orbit would affect the efficiency and capture of the organisms. Considerations include changes in the hydrostatic pressure of the bioreactor and cosmic radiation. While CB bioreactors are strong areas of research and could be used to supplement vessels’ existing carbon dioxide scrubbers, further optimization of the reactors and characterization of such bioreactors in extraterrestrial conditions must be conducted before such technology is ready to be used as a standalone scrubber.

### Carbon Fixation for Nutrition

As stated above, the strength of CB and algae are that, depending on the species, they can provide many of the vitamins and nutrients that humans require. As stated above, while CB and algal intake of carbon dioxide is being optimized, in the best current conditions, some species intake roughly 1.7 g/L/day of CO_2_. There has been research into increasing rates of carbon dioxide fixation focused on industrial purposes, such as diols or ethanol, by altering the innate biochemical pathways [76,77]. While these products may be of use elsewhere in the mission, they are not ideal for human nutrition. As written above in “Nutrition”, while many species are great sources of protein, this is not the case for carbohydrates. Many have been made into supplements that are advertised for their anti-cancer properties and touted as cholesterol lowering. While this remains to be seen, they do provide great sources of vitamins and minerals that could fuel future crewmembers.

## 8. Stage 3: Biofuel Production

Another motivation for the development of a robust bioreactor is the ability to create useful fuels. Missions beyond near Earth orbits necessitate the conservation of existing fuel and even finding ways to create it. The Mars DRA 5.0 explains the usefulness of ISRU reactors in creating fuel. A liquid oxygen/methane combination is deemed the most attractive with the reference acknowledging missions being feasible with only an O_2_ generation requirement [1]. Of course, having an ISRU system for creating methane would alleviate further restrictions on mission mass, even if a mission is possible without it. For years, cyanobacterial bioreactors have been proposed as a viable solution for this due to their rapid growth rate, ability to organically convert environmental materials to fuel, and their adaptability [78]. A few products that have been historically of interest are gasoline substitutes, hydrogen, bioethanol, oils (biodiesel), and methane.

The enzyme that is widely used in CB for carbon fixation is RuBisCo. This enzyme has been shown to have poor fixation rates in the wild, with a lack of specificity for the CO_2_ binding site, and work is ongoing to improve productivity [79] The manipulation of a carbon sink pathway by Oliver et al. in 2015 displayed increases in the total amount of carbon fixation in CB strains at a sacrifice of efficiency [80]. They also suggested that this increase in total carbon fixation still is not the solution to efficient fuel production at the moment and that decoupling of the fixation pathways from cell growth and others might be the answer [78,80].

*S. elongatus* is one of the main species that has been investigated for the production of gasoline substitutes. Though progress has been made to produce ethanol effectively, some focus has shifted to finding solutions to produce a compound that has higher energy density to match with our current infrastructure [78]. The table below (Table 4) from Nozzi et al. shows the summary of their research gathering for strains of the *Synechocystis* species, the compounds they produced, and the rate at which they produced them [78]. Isobutanol has been investigated as an alternative due to its increased energy density and efficiency. *S. elongatus* PCC 7942 was altered to divert the pyruvate stores to be converted to isobutyraldehyde, which can be harvested relatively easily and converted to isobutanol with a high yield due to a high vapor pressure [81]. 

**Figure 4:**
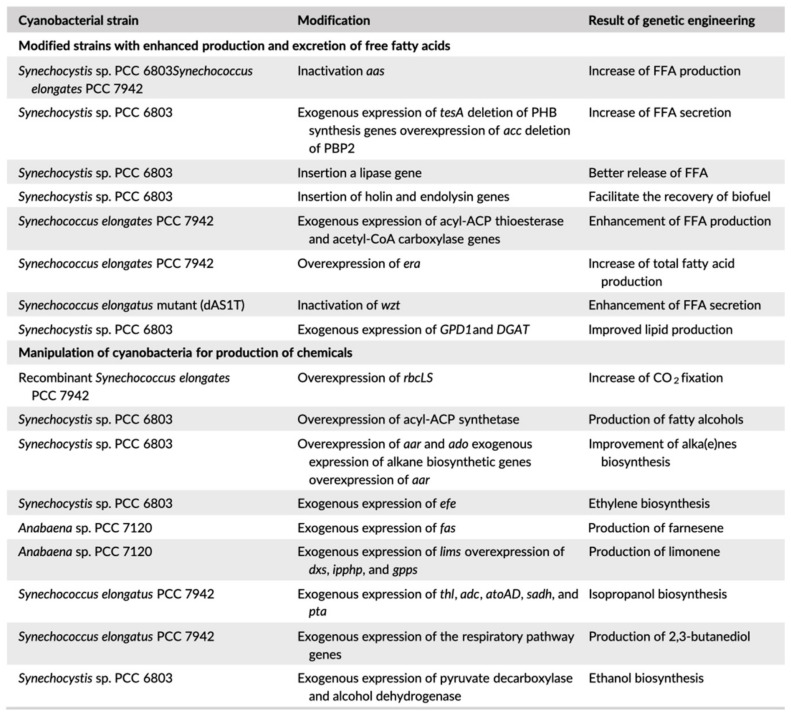
A summary of the genetic engineering studies which were focused on the improvement of cyanobacterial strains for biofuel production [82].

CB lipid profiles provide a platform of investigation for biodiesel production. High concentrations of useful fatty acids, including palmitic (C16:0) and stearic acid (C18:0), point to their potential. Indeed, higher concentrations of these specific fats over others are more favorable. Figure 4 shows a series of genetic modifications performed on multiple strains to achieve more favorable results in lipid production for biodiesel [82]. Oils can be isolated from cultures and combusted in compatible generators. This is of interest in terrestrial applications specifically because the exhausted CO_2_ will match with fixation into fatty acids, with no net addition to the atmosphere. Multiple species have been evaluated for their lipid profiles and it was found that the *Anabaena*, *Synechocystis*, *Microcystis*, and *Trichomus* species are ideal [82]. Analysis on the extraction methods from the CB biomass showed that chemical extraction by chloroform and methanol produced the highest yield [82]. However, chemical means of extraction are not ideal for use in remote deployments where they are not replenished. As far as physical methods are concerned, microwave and pulsed electric field systems were the most economical for large-scale extraction from *Synechocystis*. Currently, there is no major adoption of this system because cultivation has not been economical to this point. Given the presence of these fatty acids across the board, biodiesel opens the possibility of harvesting a fuel at many stages.

Hydrogen is another fuel product that CB are able to produce. However, production is not significant enough to provide any meaningful product for use due to the oxygen sensitivity of hydrogenases [81]. Sadvakasova et al. also wrote a comprehensive review of the literature surrounding the bioproduction of hydrogen in 2020. Among their conclusions, they showed that experiments have been run to optimize hydrogen production and there is promise in genetic alteration of the CB for more favorable gene expression combinations, but that at this time, hydrogen production has not been made effective nor efficient enough for large-scale implementation [83].

Among proposals for ISRU bioreactors, methane has been one of the bigger players. The ideal goal is to have a system that converts CO_2_ to CH_4_ while also having a low energy requirement, no water hydrolyzing facility, and no catalyzer. Yeung et al. tested the relative methane production of four reactors, with CB, methanogens, or both. One of the bioreactors, containing both planktonic and biofilm CB as well as methanogens and stored in the dark, produced methane at a 3.5 times higher rate than just CB or the methanogens [84]. Production increased over a short-term period before biofilm production likely prevented the degradation community from accessing the proper nutrients [84].

An approach to the two reactor model was tested in 2013 when phosphorus-starved *A. platensis* was used as food for anaerobic digestion into methane production. When starved of phosphorus, *A. platensis* becomes enriched in carbohydrates. The results showed that increasing the carbohydrate input yielded higher methane production, up to 66% more with heat pretreated biomass [85]. Further, it was noted that freezing the *Arthrospira* biomass might have a positive effect on biodegradability, which also complements our discussion on optimal storage techniques [85]. In another instance, the *Arthrospira*, *Chlorella*, and *Anabaena* species were each cultivated in photobioreactors and then used as inputs into anaerobic bioreactors for biogas production (Table 5) [86]. *Chlorella* demonstrated the fastest growth of dry weight among the three, leading to it producing the highest rate of biogas production. Specifically, 95% pure methane was achieved through the use of a permabsorber module. Their compared production rates are shown below. In 2012, *Chlorella* was again tested for methane production as an input for anaerobic digestion and this study found that methane production increased with increasing loading dose into the anaerobic digester up to a saturation point, leading to a maximum methane production rate of 100 ± 25 mL/L/day [87]. If this process is made more efficient and scaled up in size, methane production could be significant enough for use. While not historically favored, *Chlorella* deserves a harder look into viability for the phototrophic biomass producer in the systems designed for space travel.

Pairing a photosynthetic reactor with a methanogenic reactor has been proven to work, however, it still needs to be investigated and optimized further. Building on the current state of research is key to making this concept a reality on-board a spacecraft and on the ground. Another note, when establishing an ISRU/BLSS system on another celestial body, one must be conscious about where the carbon is being stored. If you are on a carbon-rich planet, such as Mars with its atmosphere, then there is plenty of carbon to use. However, when on the moon or a carbon-poor location, it is important to not sequester all of your carbon supply in methane fuel because then you will not have enough to cycle back into the photosynthetic reactor.

## 9. Application for Earth

The pursuit of spaceflight has brought significant spin-off technology for terrestrial use. It has consistently been an arena of incredible scientific innovation over the last century. Attempting to support human life in an environment so austere and limiting requires a reimagining of every living process we take for granted on Earth. Water filtration systems had to be developed due to the expense of frequent resupply missions. Complex computer systems were needed to make complex calculations in fractions of a second. LASIK procedures came from contributions from the effort to use lasers used for docking accuracy. A vast amount of technology that we use today was developed in part by efforts at NASA and its partners, from thermal insulation to impeller pumps used in ventricular devices. Continuing to push bigger and bolder goals will advance our capacity to bring efficient and effective countermeasures to real human problems into existence for the whole world to use. 

The goal of the initiative mentioned in this paper is the development of bioreactors that fulfill nutritive, gaseous, and fuel-related shortcomings for humans in remote environments. CBs and microalgae can be a rich source of proteins and other nutrients. Optimizing the creation of bioreactors for human consumption could be an effective solution to malnutrition worldwide. Specifically, kwashiorkor, a severe malnutrition defined by adequate calories but inadequate protein consumption resulting in peripheral pitting edema, muscle atrophy, and distended abdomen, is responsible for 6 million deaths annually in underdeveloped and developing countries, and the rate is expected to continue to rise [21]. Wide distribution of bioreactors could provide the protein source needed in these countries to prevent protein energy malnutrition. While not always an excellent source of carbohydrates as mentioned previously, harvests from these reactors could supplement the crops and other native foods where they are deployed on Earth. 

If reactors that include a fuel producing component in addition to the nutritive component are deployed, then societies beyond traditional trade routes could then access fuels for vehicles and electricity, given that they have compatible infrastructure. Once space agencies develop bioreactors that can fit in the confines of a spacecraft, the size of these reactors would likely then be small enough for easy mobility around the globe. If we can get one to a planet millions of miles away, dropping one off in any number of developing countries would be physically feasible barring any financial barriers. Renewable nutrition, fuel, and oxygen supplies would revolutionize the socioeconomic development of the world. 

## 10. Conclusions

Over the last decade and half, the science of exploring the use of ISRU/BLSS bioreactors has advanced tremendously. New siderophilic strains with improved bioweathering capacity continue to be discovered. Many species have been characterized by their ability to provide a portion of a human diet, as well as provide a breathable atmosphere and propellant to continue missions. From a nutrition standpoint, *Arthrospira* is a genus that has been most studied and which has secondary benefits that cannot be refuted. Other species provide similar benefits as well and should not be discounted. For atmospheric control, from the research, oxygen production has been effectively seen in many bioreactors in the past and can sustain human needs in space. The optimization of carbon fixation processes should be further investigated. Enzyme specificity and efficiency are included in the current roadblocks, but gene manipulation shows hope for a better solution. Fuel production is a mixed story. Ethanol does not have the energy density of other compounds. Sustainable hydrogen production has not been effectively developed. Biodiesel/gasoline are not optimal for rocket engines. However, methane is a fuel that could be employed for an ascent engine, and perhaps the Mars–Earth trans-injection burn, and it shows promise for large-scale ISRU production. When used with the oxygen byproduct of the photosynthetic reactor, this makes extra-terrestrial fuel production a real possibility. Promising species have been identified for each part of a complete system, but there is always room for improvement. Models of complementary photosynthetic–methanogenic bioreactors have shown compatibility to a degree, but should still be improved for efficiency. Though we do not have access to large amounts of lunar or Martian regolith now, analogs have been made to jumpstart trials of biological ISRU systems. This field is advancing fast and once solved, not only will we exceed the current limits on space travel, but also shape the distribution of renewable food and energy here on Earth.

## Figures and Tables

**Table 1:** Essential amino acid requirements for adult humans (Data from the National Academy of Sciences) [16].

Amino Acid	Daily Requirement per Adult Human in mg/g
Histidine	8–12
Isoleucine	10
Leucine	14
Lysine	12
Methionine plus cysteine	13
Phenylalanine plus tyrosine	14
Threonine	7
Tryptophan	3.5
Valine	10

**Table 2:** The total proteins and antioxidant molecules in *A. platensis* biomass fresh and differently processed [31].

Biomolecules	*A. platensis*	*A. platensis*	*A. platensis*	*A. platensis*
	Fresh	Frozen	Oven-Dried	Freeze-Dried
**Total Proteins** **(mg g D.W.^−1^)**	188.60 ± 13.55 b	283.96 ± 11.79 a	122.73 ± 2.53 c	167.09 ± 4.35 b
**Ascorbic acid** **(mg g D.W.^−1^)**	1678.29 ± 2.62 b	3149.54 ± 7.99 a	354.79 ± 0.93 d	1403.9 ± 11.49 c
**Dehydroascorbic acid** **(mg g D.W.^−1^)**	1998.99 ± 7.01 c	1362.93 ± 22.78 d	3296.69 ± 15.56 b	4660.74 ± 34.56 a
**Total Phenols** **(mg g D.W.^−1^)**	15.77 ± 1.10 b	22.65 ± 0.46 a	12.14 ± 1.84 c	11.91 ± 0.28 c
**Total Flavonoids** **(mg g D.W.^−1^)**	20.82 ± 0.08 b	8.04 ± 0.29 c	4.31 ± 0.11 d	30.92 ± 0.17 a

Data are expressed as the mean ± s.e. (standard error). Different letters, in the same row, indicate significant differences (*p* ≤ 0.05).

**Table 3:** The phenolic compounds in *A. platensis* samples [31].

Compounds	Rt	*A. platensis*	*A. platensis*	*A. platensis*	*A. platensis*
		Fresh	Oven-Dried	Frozen	Freeze-Dried
Gallic acid	6.11	+	+	+	+
Catechin	11.28	+	+	+	-
Caffeic acid	13.22	+	-	+	-
*p*-Hydroxybenzoic acid	14.13	+	+	+	-
*p*-Cumaric acid	18.69	+	+	-	+
Ferulic acid	18.81	+	+	-	+
Quercetin	29.59	+	-	+	+
Genistein	34.95	+	+	+	+
Kaempferol	36.67	+	+	+	+

Rt, retention time; +, compound detected; -, compound not detected.

**Table 4:** Titers for various biochemicals [78].

Compound	Organism	Titer
Acetone	*Synechocystis* sp. PCC6803	36 mg/L
2,3-Butanediol	*S. elongatus* sp. PCC7942	2.4 g/L
1-Butanol	*S. elongatus* sp. PCC7942	30 mg/L
Ethanol	*Synechocystis* sp. PCC6803	5.5 g/L
Ethylene	*Synechocystis* sp. PCC6803	171 mg/L⋅day
Fatty acids	*Synechocystis* sp. PCC6803	197 mg/L
Isobutanol	*S. elongatus* sp. PCC7942	450 mg/L
Isobutyraldehyde	*S. elongatus* sp. PCC7942	1.1 g/L
Isoprene	*Synechocystis* sp. PCC6803	50 μg/g dry cell⋅day

**Table 5:** Comparison of the productivity for the selected producers with biogas output after anaerobic digestion of the phototrophic biomass [86].

Organism	Biomass Productivity	Biogas Output
	(g/L Day)	(mL/L Day)
*S. platensis*	0.25	125
*A. variabilis*	0.66	300
*Chlorella* sp.	1.00	300

## Data Availability

No new data were created or analyzed in this study. Data sharing is not applicable to this article.

## Image Copyright

All images, figures, and tables were obtained and approved for re-use through either permission from their original authors, publishers, or through a Creative Commons license.
